# Healthcare utilization patterns and costs related to neurofibromatosis 1 in Ontario, Canada

**DOI:** 10.1186/s13023-025-04166-5

**Published:** 2026-01-26

**Authors:** Ajith Sivadasan, Alejandro Hernandez, Elisa Candido, Patricia C. Parkin, Karen Tu, Meg Mendoza, Carolina Barnett-Tapia

**Affiliations:** 1 Elizabeth Raab Neurofibromatosis Clinic, University Health Network, Toronto, Canada; 2 ICES, Toronto, Canada; 3 Pediatric Neurofibromatosis Clinic, Department of Pediatrics, The Hospital for Sick Children, University of Toronto, Toronto, Canada; 4 Department of Family and Community Medicine, Toronto Western Family Health, University of Toronto, North York General Hospital, Team-University Health Clinic, Toronto, Canada; 5 Division of Neurology, Department of Medicine, University of Toronto, 200 Elizabeth ST. 5EC Rm 334, Toronto, ON M5G 2C4 Canada

**Keywords:** NF1, Healthcare utilization, Health-care costs

## Abstract

**Background and objectives:**

Neurofibromatosis type 1 (NF1) is a multisystemic disease, characterized by cutaneous manifestations and peripheral nerve sheath tumors. Patients also have a high prevalence of learning disability, gliomas, as well as other malignancies, and require specialized follow up and surveillance. However, there are limited data regarding how people with NF1 use the healthcare system. We aimed to assess the use of different health services in individuals with NF1 compared to the general population.

**Methods:**

This population-based, matched cohort study in Ontario, Canada, used a registry of individuals with confirmed NF1 from pediatric and adult clinics between 1990 and December 31, 2020, linked to administrative health databases. Each patient was matched 1:5 to population controls, by date of birth, sex, income quintile and geographic area of residence. We compared outpatient primary and specialty claims, hospitalizations, emergency department (ED) visits, same-day surgeries, overall healthcare costs and use of disability benefits.

**Results:**

1,210 individuals with NF1 were matched to 6,050 controls, mean follow up was 19.6 ± 8.7 and 18.8 ± 8.5 years, respectively; at the end of the study window, mean age was 26.2 ± 16.9 years. More adults with NF1 received disability benefits than controls (17.6% vs. 6.6%, *p* < 0.001). NF1 individuals had more ED visits (RR:1.11, 95% CI: 1.04–1.19), hospitalizations (RR: 2.66, 95% CI: 2.43–2.91), primary care visits (RR: 1.15, 95% CI: 1.10–1.21), specialist visits (RR:2.34, 95% CI: 2.18–2.51) and same-day surgeries (RR: 1.62, 95% CI: 1.47–1.69). Healthcare costs were higher in NF1 than controls (CAD$53,858 vs. CAD$18,133, *p* < 0.0001). Individuals with NF1 in rural areas had more ED visits and fewer primary and specialty visits than urban dwellers; those in the highest income quintile had fewer ED visits and hospitalizations.

**Discussion:**

Individuals with NF1 in Ontario, Canada, have high use of outpatient and inpatient services, disability benefits and higher healthcare costs, highlighting the need for multidisciplinary care. Rurality and income quintile were associated with the use of healthcare resources; future work is needed to assess social determinants of health in NF1.

**Supplementary Information:**

The online version contains supplementary material available at 10.1186/s13023-025-04166-5.

## Background

Neurofibromatosis Type 1 (NF1) is one of the commonest autosomal dominant conditions with estimated prevalence of 1/1900 to 1/3500 [1]. NF1 is caused by pathogenic variants in the NF1 gene [2]. The diagnostic criteria, updated in 2021, are highly sensitive and specific in patients with NF1 [3, 4]. There is marked phenotypic heterogeneity even within families [5]. 

NF1 is a multisystem disease, with common cutaneous manifestations such as cutaneous neurofibromas which cause pain and disfigurement [6]. Other comorbidities include malignancies, central nervous system tumors, angiopathy (renal artery stenosis, Moya-Moya disease), osteoporosis and learning disabilities [7]. The risk of malignant peripheral nerve sheath tumor (MPNST) is estimated around 6–15% in a lifetime [6]. Additionally, individuals with NF1 have higher risk of other malignancies, like breast cancer and pheochromocytoma, and have a 8–15 year reduction in the average life expectancy [8–11]. 

Periodic surveillance of people with NF1 can potentially reduce morbidity and mortality. A multidisciplinary approach is needed to improve access to care, facilitate timely referrals and transition to adult care. However, there are limited population-based data, with scarce data on health utilization patterns, access to care, associated costs and health outcomes at the population level. This population-based data is important in identifying the burden of the disease and devising strategies regarding unmet needs and the need for effective interventions. Our aim was to study how individuals with NF1 use the healthcare system compared to population controls, including the number of primary care, specialist, emergency visits, outpatient surgeries, hospital admissions, mortality and overall healthcare associated costs.

## Methods

### Patients

The Hospital for Sick Children’s Pediatric NF clinic was established in the 1990s. The clinic is composed of pediatricians, with referrals to other specialties as needed. When adolescent patients turn 18 years of age, they are transferred to adult care. The Elisabeth Raab adult Neurofibromatosis clinic at the University Health Network (UHN) was established in 2015. The core clinic is composed of several specialties, including neurology, neurosurgery, family medicine and medical genetics; patients are referred to other specialties as needed. The registry includes individuals with confirmed NF1 seen at either clinic since clinic inception until December 2020. The Research Ethics Boards of the Hospital for Sick Children and UHN approved the retrospective registries and linkage to administrative data.

In Ontario, ICES holds a repository of all administrative health data in the province. ICES is an independent, not-for-profit research institute, whose legal status under Ontario’s health information privacy law allows it to collect and analyze health care and demographic data without consent, for health system evaluation and improvement. The use of the administrative data in this project is authorized under section 45 of Ontario’s Personal Health Information Protection Act (PHIPA) and does not require review by a Research Ethics Board. This report follows the STROBE reporting guideline for observational studies.

In Ontario, virtually all the population is covered through the publicly funded Ontario Health Insurance Plan (OHIP), and each eligible individual has a unique OHIP number. ICES databases used in this study include the registered Persons Database (RPDB), the Ontario Health Insurance Plan (OHIP) database, the National Ambulatory Care Reporting System (NACRS), the Canadian Institutes of Health Information Same Day Surgery (SDS) database, the Canadian Institutes of Health Information Discharge Abstract Database (DAD) and the Ontario Drug Benefit Claims (ODB) database. A full list of all databases used is in the Supplementary material. These datasets were linked using unique encoded identifiers and analyzed at ICES. In addition to the clinic registries, we also linked individuals with confirmed NF1 identified though chart review of primary care electronic medical records for a previous study of NF1 in Ontario [12]. After linkage we removed any duplicates.

Because NF1 is a genetic condition, to capture longitudinal healthcare utilization, the index date was defined as the date of birth or first date of OHIP eligibility (i.e. immigration) for persons born on or after January 1, 1992. For persons born before January 1, 1992, index date was January 1st, 1992, or the first date of OHIP eligibility. Exclusion criteria were not living in Ontario at index date, less than 2 years of OHIP eligibility (beyond index date) and missing values for matching variables. The eligible individuals were matched 1: 5, without replacement, to general population controls. Matching was based on index year, age, sex, neighborhood income quintile and rurality. Individuals were followed until any of the following: (1) date of death for any cause, (2) date of OHIP eligibility loss (e.g. emigration) or (3) end of the study window (December 31, 2020).

### Variables and outcomes

We assessed common comorbidities, including diabetes, hypertension, cancer, dementia, heart failure, and chronic obstructive pulmonary disease (COPD). We used the Johns Hopkins ACG^®^ System Version 10 to compare ACG^®^ System Aggregated Diagnosis Groups (ADG) scores, which is weighted score representing the presence or absence of the 32 diagnosis groups [13]. The ADG has been validated to predict mortality in the general adult population, through the Mortality Risk Score (MRS), which was calculated by integrating the age, sex and ADG scores [14]. 

We compared inpatient health care utilization between NF1 individuals and controls, including: emergency department (ED) visits, hospital admissions and same day surgeries. We also assessed primary care and overall specialist visits, as well as specific specialties, including neurology, neurosurgery pediatrics, genetics, dermatology, plastic surgery, medical oncology, general surgery, ophthalmology and psychiatry. We obtained healthcare costs associated with hospital admissions, ED visits, same day surgery, outpatient costs, physicians claims and drugs costs. Costing data includes services between 2007 and 2020 and are standardized to 2020 Canadian dollar [15]. 

Outpatient prescriptions are only publicly funded in Ontario for seniors aged 65 years and older, and select populations including persons aged 25–64 years receiving social assistance such as disability benefits. Within the NF1 individuals and controls who were eligible for disability benefits at any point during the study window, we compared the proportion who received disability benefits, using the Ontario Drug Benefit claims.

Finally, we assessed the number of deaths in the NF1 and control groups during the study window, and we compared the proportion of deaths and the mean age of death.

### Analyses

The statistical analysis was performed with SAS EG version 8.3. Continuous variables are represented as mean (standard deviation) and categorical variables as number (percentage). We used standardized mean difference (SMD) to compare mean/ median values and proportions between NF1 and controls, whereby SMD ≥ 0.1 indicates a significant difference between groups. The annual rates of use (in person-years) were calculated by dividing the number of encounters by the total time of follow up of the population-at-risk. The risk ratio was calculated by dividing the annual rate of encounters for NF1 patients by the rate in controls; this was adjusted for age, sex, income, and comorbidities using binomial regression. A risk ratio > 1 indicates higher utilization in NF1 patients.

To assess factors associated with healthcare use within NF1 individuals, we performed negative binomial regression in the NF1 cohort (excluding controls), adjusting for age, sex, income, and comorbidities, for each of the main outcomes (ED visits, hospitalizations, same day surgery, primary care visits and specialty visits).

## Results

**Fig. 1:**
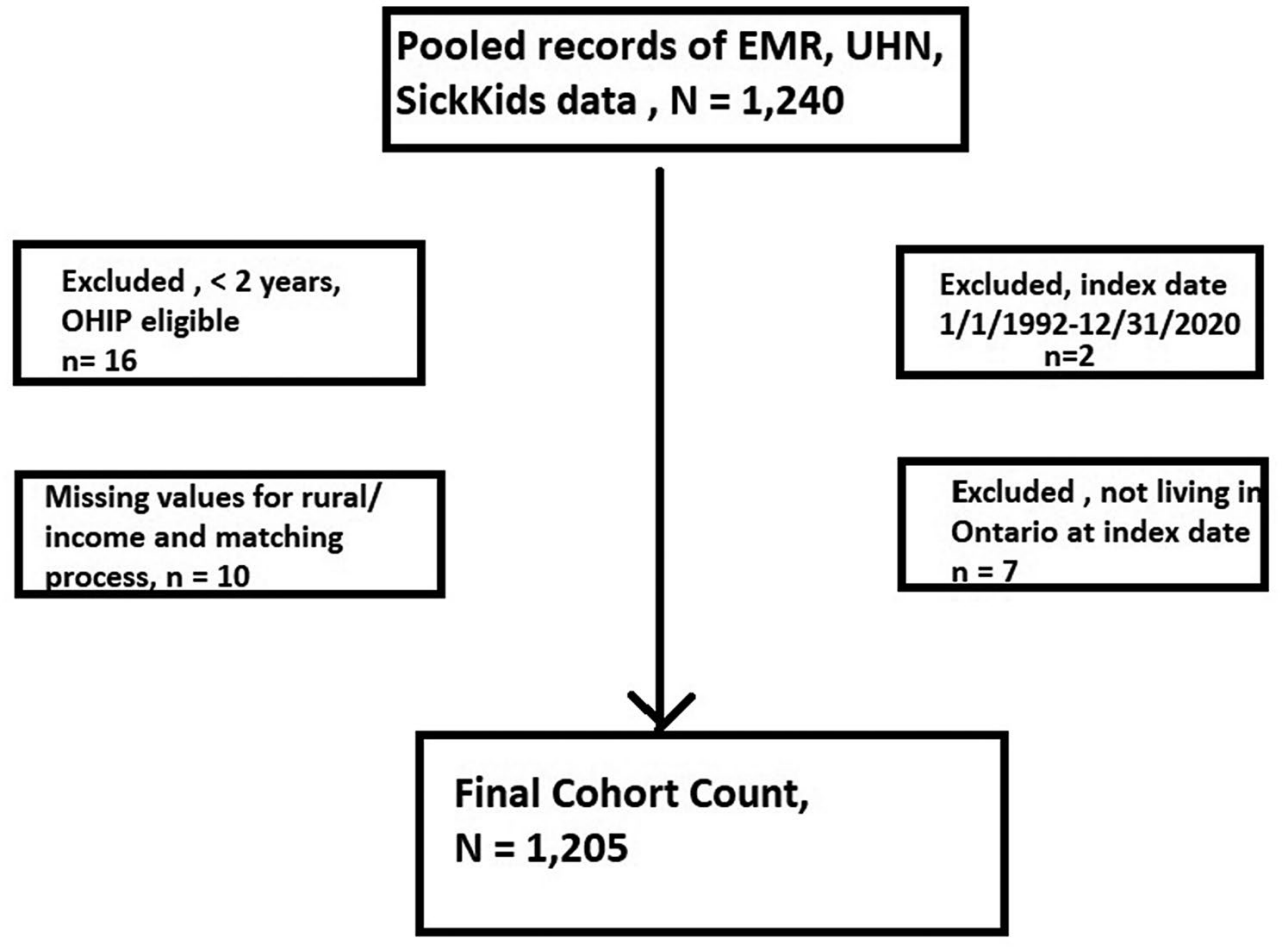
Consort Diagram of the NF1 Cohort Creation

There were 592 NF1 patients identified from the Hospital for Sick Children, 720 from UHN and 71 from the EMR study. Out of the total 1,383 records, 143 were excluded as they were duplicates. Of the 1,240 individuals with NF1, 1,210 met the inclusion criteria (Fig. 1) and these were matched to 6,050 controls. The mean follow-up time was 19.6 ± 8.7 years in NF1, and 18.7 ± 8.5 years in controls, with 23,772 and 113,156 person-years, respectively. At the end of the study window, the mean age in the NF1 group was 26.7 ± 17.6 years (median 23, IQR: 13–37), and 613 (50.7%) were female. From 544 individuals with NF1 and 2,677 controls aged 25–64 years during the study window, a higher proportion of NF1 individuals received disability benefits than controls (17.5% vs. 6.3%, SMD:0.35) based on the ODB data. During the study window, there were 21 (1.7%) deaths in the NF1 group, compared to 111 (1.8%, p = ns) in the controls; there was no difference in the mean age of death (47.9 ± 24.3 vs. 45.8 ± 21.5) between groups.

More individuals with NF1 had a diagnosis of hypertension (8.7% vs. 6.1%, SMD = 0.1) and cancer (11.7% vs. 2.3%, SMD:0.37) compared to the controls. The mean ADG weighted score and mortality risk scores were significantly higher in the NF1 cases compared to the controls. The full demographic and clinical variables are in Table 1.

**Table 1:** Demographics at the end of the observation window

Variable	NF 1 cases (*N* = 1210)	Controls (*N* = 6050)	SMD
**Age (mean**,** SD)**	26.7 ± 17.6	26.1 ± 16.8	0.038
**Age categories (n**,** %)**			
0–9 years	182 (15.0%)	887 (14.7%)	0.011
10–19 years	324 (26.8%)	1641 (27.1%)	0.008
20–29 years	290 (24.0%)	1566 (25.6%)	0.044
30–39 years	145 (12.0%)	724 (12.0%)	0.001
40–49 years	110 (9.1%)	530 (8.8%)	0.012
50–59 years	88 (7.3%)	408 (6.7%)	0.021
60–69 years	42 (3.5%)	184 (3.0%)	0.024
≥ 70 years	29 (2.4%)	110 (1.8%)	0.054
**Sex (n**,** %)**			
Female	613 (50.7)	3065 (50.7%)	0
Male	597 (49.3%)	2985 (49.3%)	0
**Urban Residence**	1143 (94.5%)	5423 (89.6%)	0.18 *
**Area income quintile (Q)**			
Q1 (lowest)	252 (20.8%)	1159 (19.2%)	0.04
Q2	237 (19.6%)	1152 (19.0%)	0.01
Q3	211 (17.4%)	1156 (19.1%)	0.04
Q4	255 (21.1%)	1181 (19.5%)	0.04
Q5 (highest)	250 (20.7%)	1296 (21.4%)	0.02
**Comorbidities**,** n %**			
Diabetes	31 (2.6%)	196 (3.2%)	0.04
Hypertension	105 (8.7%)	362 (6.0%)	0.10
Cancer	141 (11.7%)	183 (3.0%)	0.33 *
Dementia	7 (0.6%)	12 (0.2%)	0.06
Heart Failure	14 (1.2%)	28 (0.5%)	0.08
COPD	37 (3.1%)	144 (2.4%)	0.04
**ADG score**,** Mean (SD)**	10.73 (12.1)	2.69 (8.8)	0.76 *
**MRS**,** Mean (SD)**	18.02 (19.8)	13.98 (16.7)	0.22 *
**Disability Benefits §**	95 (17.5%)	169 (6.3%)	0.35 *

ADG: Aggregated Diagnosis Groups MRS: Mortality Risk Score

§ There were 544 individuals with NF1 and 2,677 controls aged 25–64 years during the study window, who would be eligible for disability benefits

SMD: Standardized Mean Difference, ≥ 0.1 indicates significant differences between groups

The proportion of patients with any ED visit, hospitalization, same day surgery, primary and specialty visits was higher in NF1 than controls. Overall, NF1 patients had more visits to the ED (adjusted RR:1.11, 95% CI: 1.04–1.19), hospital admissions (adjusted RR: 2.66, 95% CI: 2.43–2.91), primary care visits (adjusted RR: 1.15, 95% CI: 1.10–1.21), specialist visits (adjusted RR:2.34, 95% CI: 2.18–2.51) and same-day surgeries (adjusted RR:1.62, 95% CI: 1.47–1.79), as shown in Table 2.

**Table 2:** Health care utilization outcomes

Outcome variable	NF 1 cases (*n* = 1,210) 23,672 person years	Controls (*n* = 6,050) 1,13,181 person years	*p*-value	Rate Ratio (95% CI)
**ED visits**				
Yes, n (%)	1,096 (90.6%)	4,956 (81.9%)	< 0.0001	
Mean number of visits (SD)	8.8 (11.8)	6.8 (8.5)	< 0.0001	
Rate of use	0.41	0.30		1.11 (1.04–1.19)
**Hospitalizations**				
Yes, n (%)	999 (82.6%)	2,065 (34.1%)	< 0.0001	
Mean number of visits (SD)	3.02 (3.39)	2.34 (2.98)	< 0.0001	
Rate of use	0.13	0.04		2.66 (2.43–2.91)
**Primary care visits**				
Yes, n (%)	1,179 (97.4%)	5,828 (96.3%)	0.0552	
Mean number of visits (SD)	70.35 (78.14)	51.76 (54.00)	< 0.0001	
Rate of use	3.49	2.67		1.15 (1.10–1.21)
**Specialist visits**				
Yes, n (%)	1,205–1,209 (99%)	5,378 (88.9%)	< 0.0001	
Mean number of visits (SD)	70.96 (65.90)	26.97 (39.50)	< 0.0001	
Rate of use	3.61	1.28		2.34 (2.18–2.51)
**Same-day surgery visit**				
Yes, n (%)	663 (54.8%)	2,168 (35.8%)	< 0.0001	
Mean number of visits (SD)	3.42 (3.88)	2.39 (3.42)	< 0.0001	
Rate of use	0.10	0.05		1.62 (1.47–1.79)

ED: Emergency Department

Rate Ratio through binominal modelling adjusted by age, sex, income quintile, rurality and ADG. Specialty visits presented as range, to minimize risk of identification, given low number of individuals with NF1 without specialty visits

Mean number of visits on those individuals with ≥ 1 visit during the study window

When assessing the effect of age, sex, income quintile, rurality and ADG scores on the primary outcomes within the NF1 population only (excluding controls), we found that sex was not associated with any outcome. Age had a marginal relationship with healthcare visits, whereby higher age was associated with fewer ED visits, hospitalizations and specialty visits (RRs between 0.98 and 0.99), but with slightly more primary care and same day surgeries (RRs between 1.01 and 1.02). Income quintile was strongly associated with rate of ED visits: individuals in the lowest income quintile had more visits than individuals in all other quintiles. Individuals with NF1 in the highest income quintile had fewer hospitalizations (RR 0.82, 95% CI: 0.69–0.98) and primary care visits (RR: 0.80, 95% CI: 0.70–0.91) than those in the lowest quintile. Individuals with NF1 in rural areas had more visits to the ED (RR: 1.92, 95% CI: 1.40–2.36), as well as fewer primary care (RR: 0.73, 95% CI: 060-0.89) and specialist visits (RR: 0.78, 95% CI: 0.66–0.93). The ADG score was associated with marginally higher rates of all healthcare use (RR between 1.01 and 1.04). Table 3 shows the details for each outcome.

**Table 3:** Adjusted rate ratios for healthcare utilization within the NF1 cohort (*n* = 1,210)

Covariates	ED Visits RR (95% CI)	Hospitalizations RR (95% CI)	Same-day surgery RR (95% CI)	Primary Care visits RR (95% CI)	Any Specialist Visits RR (95% CI)
Age	**0.99 (0.98–0.99)**	**0.98 (0.98–0.98)**	**1.02 (1.02–1.03)**	**1.01 (1.01–1.01)**	**0.99 (0.98–0.99)**
Female (vs. Male)	1.05 (0.93–1.18)	0.99 (0.89–1.11)	1.07 (0.93–1.24)	1.18 (1.08–1.29)	1.05 (0.97–1.13)
Area Income Quantile (Q)					
Q 2 vs. 1	**0.78 (0.65–0.94)**	0.88 (0.74–1.05)	1.03 (0.82–1.29)	0.90 (0.79–1.03)	1.00 (0.89–1.13)
Q 3 vs. 1	**0.68 (0.56–0.83)**	0.87 (0.72–1.04)	1.11 (0.88–1.40)	0.99 (0.86–1.14)	1.01 (0.89–1.14)
Q 4 vs. 1	**0.69 (0.58–0.83)**	0.86 (0.73–1.03)	1.06 (0.84–1.33)	0.89 (0.78–1.02)	1.00 (0.89–1.13)
Q 5 vs. 1	**0.56 (0.47–0.68)**	**0.82 (0.69–0.98)**	1.07 (0.86–1.35)	**0.80 (0.70–0.91)**	0.98 (0.87–1.11)
Rural (vs. urban)	**1.82 (1.40–2.36)**	1.15 (0.90–1.47)	1.15 (0.84–1.58)	**0.73 (0.60–0.89)**	**0.78 (0.66–0.93)**
ADG score weighted	**1.03 (1.02–1.03)**	**1.04 (1.03–1.04)**	**1.02 (1.02–1.03)**	**1.01 (1.00-1.01)**	**1.03 (1.02–1.03)**

RR: rate ratio. Bolded values are those with significant difference in rate ratios

Area Income Quantile: Q1 having the lowest and Q5 the highest income

Individuals with NF1 had significantly more visits to all specialties than controls. The specific specialties consulted by a highest proportion of NF1 individuals were: ophthalmology (89.1%), pediatrics (83.4%), neurology (60.1%), dermatology (52.4%) and genetics 566 (46.9%). Table 4 shows the proportion of individuals with NF1 and controls and specialty clinic visits.

**Table 4:** Use of specialty Services, NF1 and controls

Specialty	NF 1 cases (*N* = 1210)	Controls (*N* = 6050)	*P* value
**Ophthalmology**			
Yes, n (%)	1,078 (89.1%)	1,270 (21.0%)	< 0.0001
Mean number of visits (SD)	8.59 (8.89)	4.89 (8.03)	< 0.0001
**Pediatrics**			
Yes, n (%)	1,009 (83.4%)	3,373 (55.8%)	< 0.0001
Mean number of visits (SD)	31.02 (34.79)	14.69 (21.71)	< 0.0001
**Neurology**			
Yes, n (%)	727 (60.1%)	615 (10.2%)	< 0.0001
Mean number of visits (SD)	6.79 (9.35)	3.23 (4.45)	< 0.0001
**Dermatology**			
Yes, n (%)	634 (52.4%)	1,827 (30.1%)	< 0.0001
Mean number of visits (SD)	4.59 (5.48)	4.73 (8.03)	0.6888
**Genetics**			
Yes, n (%)	567 (46.9%)	51 (0.8%)	< 0.0001
Mean number of visits (SD)	1.66 (1.21)	2.00 (1.46)	0.0597
**Plastic surgery**			
Yes, n (%)	488 (40.3%)	961 (15.9%)	< 0.0001
Mean number of visits (SD)	6.74 (8.15)	3.16 (3.50)	< 0.0001
**Neurosurgery**			
Yes, n (%)	488 (40.3%)	139 (2.3%)	< 0.0001
Mean number of visits (SD)	6.47 (6.67)	2.61 (2.69)	< 0.0001
**General surgery**			
Yes, n (%)	440 (36.4%)	1,223 (20.2%)	< 0.0001
Mean number of visits (SD)	6.42 (10.19)	4.06 (5.81)	< 0.0001
**Psychiatry**			
Yes, n (%)	301 (24.9%)	952 (15.7%)	< 0.0001
Mean number of visits (SD)	16.49 (42.70)	16.06 (46.93)	0.8879
**Oncology radiation**			
Yes, n (%)	93 (7.7%)	93 (1.5%)	< 0.0001
Mean number of visits (SD)	7.67 (7.52)	6.54 (5.55)	0.2455
**Oncology-Medical**			
Yes, n (%)	33 (2.7%)	53 (0.9%)	< 0.0001
Mean number of visits (SD)	5.06 (5.52)	9.43 (10.67)	0.0323
**Other**			
Yes, n (%)	1,049 (86.7%)	4,104 (67.8%)	< 0.0001
Mean number of visits (SD)	20.27 (31.62)	13.10 (20.37)	< 0.0001

Mean number of visits on those individuals with ≥ 1 visit during the study window

Between 2007 and 2020, individuals with NF1 had higher mean costs associated with healthcare than controls (CAD$53,858 vs. CAD$18,133, *p* < 0.0001). The largest contributor to total healthcare costs were physicians’ claims (CAD$11,993 ± 98,251) followed by inpatient costs (CAD$11,706 ± 30,514). Details can be seen in Table 5.

**Table 5:** Costs associated with healthcare Use, 2007–2020

Typo of Service	NF1 cases *N* = 1,210	Controls *N* = 6,050	SMD	*P*-value
**Inpatient costs**				
Mean (SD)	11,706.56 (30,513.88)	3,354.21 (16,381.37)	0.341	< 0.0001
Median (Q1-Q3)	972 (0–9,462)	0 (0–0)	0.72	< 0.0001
**Outpatient costs**				
Mean (SD)	8,929.32 (10,257.49)	1,719.08 (3,885.42)	0.93	< 0.0001
Median (Q1-Q3)	5,662 (2,766-11357)	381 (0-1729)	1.744	< 0.0001
**Same-day surgery costs**				
Mean (SD)	1,329.00 (2,301.59)	651.37 (1,655.27)	0.338	< 0.0001
Median (Q1-Q3)	0 (0-1849)	0 (0-492)	0.39	< 0.0001
**ED visits costs**				
Mean (SD)	1,732.00 (3,452.45)	1,067.74 (1,926.82)	0.238	< 0.0001
Median (Q1-Q3)	717 (219–1844)	506 (120-1,272)	0.252	< 0.0001
**Physician claims costs**				
Mean (SD)	11,993.32 (13,776.14)	4,528.86 (8,584.67)	0.65	< 0.0001
Median (Q1-Q3)	8,054 (4,162 − 14,590)	2216 (908-5,054)	1.169	< 0.0001
**Drugs costs**				
Mean (SD)	3,251.73 (14,911.00)	1,394.23 (12,608.36)	0.135	< 0.0001
Median (Q1-Q3)	60 (0-522)	22 (0-166)	0.31	< 0.0001
**TOTAL costs**				
Mean (SD)	53,858.00 (98,251.30)	18,132.67 (70,133.63)	0.419	< 0.0001
Median (Q1-Q3)	25,592 (12,900 − 54,122)	6,321 (2,824 − 14,701)	1.263	< 0.0001

Costs in Canadian Dollars, standardized to 2020. Drug cost only available for individuals receiving disability benefits and those > 64 years old

SMD: Standardized Mean Difference. Values ≥ 0.1 indicate significant difference between groups

## Discussion

This study highlights the overall disease burden associated with NF1, with requirements for multiple specialty clinics, in keeping with the multisystem nature of the disease. In our cohort, the NF1 patients had higher prevalence of comorbid illnesses such as cancer and hypertension, which have been previously reported [6]. 

Compared to controls, NF1 individuals had more visits to both primary care and specialists; they also had more visits to the ED, admissions to hospital and surgeries. Because most patients in the registry come from NF-dedicated clinics, it is not surprising to see a high proportion of patients with pediatrician and neurology visits, given the composition of current clinics in Toronto. Most patients with NF1 (89%) had seen an ophthalmologist, whereas this was much lower in controls. This likely reflects routine ophthalmologic surveillance in patients with NF1.

We had expected that a higher proportion of individuals with NF1 would have at least one assessment by genetics during the study window. The observed proportion (47%) is in part explained by differences in practice and clinical needs between the adult and pediatric clinics. In the adult clinic, a geneticist is part of the core clinic, and most patients are offered genetic consultation, testing and reproductive risk counselling. The practice is different in the pediatric clinic, where pediatricians order genetic testing when needed, and provide counselling; as reproductive risk counselling is usually not a major need in individuals < 18 years, there is reduced need for a formal genetics consultation in that group. However, it is also possible that individuals with NF1 that graduated from pediatric care before the adult clinic was established have limited access to genetic assessment and counselling as adults. Further work is needed to understand the gaps in care resulting from lack of transition to specialized NF1 adult providers.

We found that a higher proportion of NF1 individuals received disability benefits, almost 3 times more than controls. This is similar to a smaller (*n* = 162) cohort of adults with NF1 from the adult Neurofibromatosis clinic at Toronto General Hospital, where 16% were receiving disability benefits; although there is overlap between that cohort and the current study [16]. This proportion is also in keeping with a population-based study in Finland, where 12% of individuals with NF1 had any disability-related pension, which was much higher than general population controls [17]. This is an indicator of the societal costs and impact associated with NF1.

We explored sociodemographic factors associated with healthcare use in NF1, and we found that individuals in the lowest income quintile had higher use of the ED than all other income quintiles. This is in keeping with previous studies assessing overall ED use for any cause in different countries, with the highest use in lower income individuals [18]. Additionally, we found that those in the highest income quintile had fewer primary care, and hospital admissions compared to the lowest income quintile, without significant differences in specialty visits. Our findings could stem from better overall health in the highest income group and/or more access to educational resources regarding NF1, requiring less contact with the healthcare system. In Ontario, primary care and specialty consultations are publicly funded, but individuals in the highest income quintile have better access to uninsured or hard to access services, such as physiotherapy, mental health professionals, and private removal of cutaneous neurofibromas. Access to uninsured services could reduce the need for primary care and other services.

Finally, individuals with NF1 living in rural areas had higher use of the ED, compared to urban dwellers, but also had lower rates of primary care and specialty visits. This may be explained by a lack of access to primary care providers and specialists in rural areas, where this gap is filled by the ED.

In keeping this the higher healthcare utilization in individuals with NF1, we found elevated healthcare costs in the NF1 population compared to controls. The highest cost was due to physician claims followed by hospital admissions, and this is in keeping with the high proportion of individuals with NF1 in this cohort that required a hospital admission (82.7%). A study in France (from 2013 to 2019) also estimated hospital admissions in approximately 60% of people with NF1 [19]. The higher proportion in our study may reflect a cohort of individuals with NF1 followed at specialty clinics, who may have higher disease burden compared to those followed in other settings. Further studies are needed to understand the main cause of hospitalization, to identify opportunities for early detection and treatment that may lead to reduction in hospital admissions.

Surprisingly, we did not find a significant difference in the proportion of deaths in the study window between NF1 patients and controls, nor in the mean age of death, despite higher mortality risk scores in NF1. This may reflect the overall young cohort; at the end of the study window the mean age was 26 ± 17.6 years, indicating a large proportion of children and young adults, therefore, it is possible that longer follow up times are needed to capture differences in mortality. This is in keeping with previous literature, where median age of death in NF1 individuals was higher, ranging between 54 and 71 years-old, and reported reduced life expectancy between 7 and 25 years [9–11]. Longer follow up of the patients in this registry may help ascertain long-term mortality.

Epidemiological data are important to plan health resource allocation. There are scarce studies which have analyzed the overall health care and specialty service utilization patterns in NF1. Several studies have shown the impact of neurofibromas (cutaneous and plexiform), chronic pain, scoliosis, learning disabilities and increased risk of malignancies in individuals with NF1 [20, 21]. Multiple studies have shown the impact of NF1 manifestations in quality of life, including in Canada, where patients with NF1 had reduced quality of life compared to the general Canadian population, and this was associated to pain, anxiety, depression and perceived physical appearance [16]. Our study shows that the impact of NF1 is also reflected in the elevated healthcare needs of this population, spanning multiple specialties as well as primary care. Our findings can help with resource allocation planning as lack of appropriate disease specific and holistic care can lead to delays in diagnosis of complications related to NF1, in addition to impaired quality of life and morbidity. In a study of young adults in Australia, participants reported low access to care and researchers found that ~ 40% of them had severe NF1-related complications that were previously undiagnosed [22]. Even with access to NF1 specialized services, there may be inequalities regarding what services people with NF1 receive. A recent survey to NF1 specialists showed a wide variability (17–83%) in the awareness of, as well as agreement with, evidence-based NF1 care guidelines [23]. Future work is needed to assess the impact of guideline implementation in healthcare outcomes in NF1.

A strength of our study is the large number of patients, both adults and children, with matched controls and long follow-up window. However, most NF1 patients in this cohort are from specialty clinics, so individuals with milder forms of NF1, who may need fewer resources, are likely under-represented in this study, where only a small number were identified through primary health care databases. Additionally, both the adult and pediatric clinics are in Toronto, and while they receive patients from the whole province, distance affects the ability to travel for those individuals located farther away and this may limit the generalizability of these findings. Unfortunately, previous work to identify people with NF1 through billing codes did not accurately identify individuals with NF1 in Ontario, so we were not able to cover a larger proportion of individuals with NF1 in the province [12]. Other studies in NF1 have used insurance-based cohorts, using ICD billing codes to identify individuals with NF1, but without validation of that strategy there is a risk of misclassification [24]. In our cohort, all individuals have confirmed NF1 meeting diagnostic criteria, which is a strength of this study.

## Conclusions

Individuals with NF1 in Ontario have high healthcare utilization, associated with higher healthcare costs and higher use of disability benefits compared to controls. Pathways to ensure timely access to multidisciplinary care are needed to streamline care in this population. Rurality and income were associated with healthcare use and more studies are needed to assess how social determinants of health affect outcomes in NF1.

## Supplementary Information

Below is the link to the electronic supplementary material.

Supplementary Material 1

## Data Availability

The dataset from this study is held securely in coded form at ICES. While legal data sharing agreements between ICES and data providers (e.g., healthcare organizations and government) prohibit ICES from making the dataset publicly available, access may be granted to those who meet pre-specified criteria for confidential access, available at www.ices.on.ca/DAS (email: das@ices.on.ca). The full dataset creation plan and underlying analytic code are available from the authors upon request, understanding that the computer programs may rely upon coding templates or macros that are unique to ICES and are therefore either inaccessible or may require modification.
