# Supplementary material for: Healthcare utilization patterns and costs related to neurofibromatosis 1 in Ontario, Canada
Source: Orphanet J Rare Dis. 2026 Jan 26;21:25. doi: 10.1186/s13023-025-04166-5 (PMC12837521; doi:10.1186/s13023-025-04166-5)
Supplement: Supplementary file 1 — Supplementary Material 1 [file 13023_2025_4166_MOESM1_ESM.docx]

**Supplementary Data**

**eTable 1. Databases used in the study.**

| **Database** | **Time frame** | **Description** |
| --- | --- | --- |
| Canadian Institutes of Health Information Discharge Abstract Database (DAD) | 1988-2020 | Data for acute care institutions in Ontario. |
| Canadian Institutes of Health Information Same Day Surgery (SDS) database | 1991-2020 | Data for day surgery institutions in Ontario |
| National Ambulatory Care Reporting System (NACRS) | 2000-2020 | Data on emergency department visits, day surgery and outpatient clinic visits |
| Ontario Health Insurance Plan (OHIP) database | 1991-2020 | Physicians claims for services provided, including a diagnostic code. |
| Ontario Drug Benefit Claims (ODB) database | 1990-2020 | Claims for prescription drugs received under the ODB program. Claims are for persons >= age 65 years, or < 65 years and unemployed, have disabilities, or have high prescription  costs relative to net household income. |
| Registered Persons Database (RPDB) | 1991-2020 | Demographic information (age, sex, geographic information and vital statistics) about every individual who has ever received a provincial OHIP health card number |
| Ontario Asthma Dataset (ASTHMA) | 1996-2020 | Data on prevalent and incident cases of asthma in Ontario |
| Ontario Diabetes Database (ODD) | 1991-2020 | Data on prevalent and incident cases of diabetes in Ontario |
| Chronic Obstructive Pulmonary Disease (COPD) database | 1991-2020 | Data on prevalent and incident cases of COPD in Ontario |
| Ontario Congestive Heart Failure (CHF) database | 1991-2020 | Data on prevalent and incident cases of CHF in Ontario. The CHF database is limited to those 40 years of age or older. |
| Ontario Hypertension Database (HYPER) | 1991-2020 | Data on prevalent and incident cases of hypertension in Ontario |
| Ontario Cancer Registry | 1979-2020 | Registry of prevalent cases of cancer in Ontario, including patient demographic, cancer diagnostic and death data. |
| Drugs List (DIN) | 1990-2020 | The file contains a near exhaustive list of DINs (Drug Identification Numbers) used in Canada from 1990 forward. |
| Ontario Marginalization Index (ONMARG) (1) | 2016 | ON-MARG is a geographically (Census) based index developed to quantify the degree of marginalization occurring across the province of Ontario. It is comprised of 4 major dimensions thought to underlie the construct of marginalization: residential instability, material deprivation, dependency and ethnic diversity. |
| Statistics Canada Census (CENSUS) | 2016 | Used to identify the Census based neighbourhood income quintile |
| Local Health Integration Network (LHIN) | 2009 | Local Health Integration Networks (LHINs) are not-for-profit corporations that will be responsible for planning, integrating and funding local health services in 14 different geographic areas of the province. LHINs are intended to be the managers for health services that are delivered in hospitals, long-term care facilities, community health centres, community support services and mental health agencies. Used to identify geographic area of residence. |
| Assisted Device Program (ADP) | 2000-2020 | Ontario Ministry of Health and Long-term Care data on Ontario residents with long-term physical disabilities who have accessed personalized assistive devices. Used to compute cost outcomes (GETCOST macro). |
| Client Agency Program Enrolment (CAPE) | 1999-2020 | Data pertaining to Ontario’s Ministry of Health to help establish and maintain a list of patients enrolled with specific primary care networks. Used to compute cost outcomes (GETCOST macro). |
| Continuing Care Reporting System (CCRS) | 1996-2020 | Clinical and demographic data on residents receiving facility based continuing care services. Used to compute cost outcomes (GETCOST macro). |
| Home Care Database (HCD) | 2005-2020 | Home care services data maintained by Ontario Health. Used to compute cost outcomes (GETCOST macro). |
| National Rehabilitation Reporting System (NRS) | 2000-2020 | Client data collected from participating adult inpatient rehabilitation facilities across Canada. Used to compute cost outcomes (GETCOST macro). |
| Ontario Mental Health Reporting System (OMHRS) | 2005-2020 | Data on patients in adult designated inpatient mental health beds. Used to compute cost outcomes (GETCOST macro). |

**eTable 2. Databases and variables used to define study exposures, outcomes and covariates.**

| ***Covariates*** | **Definition** |
| --- | --- |
| Age at baseline | Age in RPDB |
| Sex | Sex in RPDB |
| Geographic area of residence at baseline | Local Health Integration Network of the individual’s postal code in RPDB |
| Long-term care residence within 2 years before baseline | Admission date to LTC in CCRS within 2 years before baseline or a claim in ODB prior to baseline indicating that the claimant is in a LTC facility |
| Rural residence at baseline | Census |
| Dissemination area income quintile at baseline | Census |
| Dissemination area ethnic diversity quintile at baseline | Ethnic Diversity Quintile in ONMARG |
| Hypertension any time | Hypertension was defined as  a) one hospital admission with a hypertension diagnosis, or  b) an OHIP claim with a hypertension diagnosis followed within two years by either an OHIP claim or a hospital admission with a hypertension diagnosis.(2)  DAD, SDS  ICD-9 diagnostic codes: 401, 402, 403 404, 405  ICD-10-CA diagnostic codes: I10, I11, I12, I13, I15  OHIP  OHIP diagnostic codes: 401, 402, 403 404, or 405 |
| Asthma any time | Asthma database was used to identify patients with asthma, based on 2 or more ambulatory care visits and/or 1 or more hospitalizations.(3)  OHIP  OHIP diagnostic code: 493  DAD  ICD-9 diagnostic code: 493  ICD-10-CA diagnostic codes: J45, J46 |
| Diabetes any time | ODD was used to identify patients with diabetes, based on 2 OHIP diagnostic codes or 1 OHIP service code or 1 DAD/SDS admission within 2 years.(4)  OHIP  OHIP diagnostic code: 250  OHIP service codes: Q040, K029, K030, K045, K046  DAD, SDS  ICD-9 diagnostic code: 250  ICD-10-CA diagnostic codes: E10, E11, E13, E14 |
| Chronic pulmonary obstructive disease (COPD) any time | COPD database was used to identify patients with COPD, based on 3 or more ambulatory care visits and/or 1 or more hospitalizations within 2 years.(5)  OHIP  OHIP diagnostic codes: 491, 492, 496  DAD  ICD-9 diagnostic codes: 491, 492, 496  ICD-10-CA diagnostic codes: J41, J42, J43, J44 |
| Cancer any time | OCR database was used to identify patients with cancer, using diagnosis date occurring within the observation period. |
| Congestive heart failure (CHF) | CHF database was used to identify patients with CHF, based on 1 NACRS, DAD, SDS, or OHIP claim and a second claim (from either) in 1 year.(6)  OHIP  OHIP diagnostic code: 428  NACRS, DAD, SDS  ICD-9 diagnostic code: 428  ICD-10-CA diagnostic codes: I500, I501, I509 |

Abbreviations: ICD-9: International Classification of Diseases, 9th Revision; ICD-10-CA: International Classification of Diseases, 10th Revision, Canada

Note that there are some minor variations from the Quan et al.^7^ paper where 5-digit ICD-9 codes were specified (DAD only use ICD-9 codes to the 4^th^ digit) or the ICD-10-CA coding scheme differs from the ICD-10 coding scheme.

Possible extra table: algorithm for the age-based Mortality Risk Score and the ADG score

| **ADGs and predictor variable** | ​​**Points assigned for calculating the Mortality Risk Score** | **Weights assigned for calculating Austin's ADG Score** |
| --- | --- | --- |
| Age (for each year over 20 years old) | 1 | -- |
| Male sex | 3 | -- |
| ADG groups |  |  |
| Time Limited: Minor | -1 | 0 |
| Time Limited: Minor - Primary Infections | 1 | 0 |
| Time Limited: Major | 6 | 6 |
| Time Limited: Major - Primary Infections | 6 | 4 |
| Allergies | -5 | -6 |
| Asthma | 2 | 0 |
| Likely to Recur: Discrete | -- | 0 |
| Likely to Recur: Discrete - Infections | -- | 0 |
| Likely to Recur: Progressive | 6 | 8 |
| Chronic Medical: Stable | -2 | 4 |
| Chronic Medical: Unstable | 8 | 12 |
| Chronic Specialty: Stable - Orthopedic | -3 | -3 |
| Chronic Specialty: Stable - Ear,Nose,Throat | -3 | 0 |
| Chronic Specialty: Stable - Eye | -3 | 3 |
| Chronic Specialty: Unstable - Orthopedic | -2 | -2 |
| Chronic Specialty: Unstable - Ear,Nose,Throat | -5 | -4 |
| Chronic Specialty: Unstable - Eye | -2 | 1 |
| Dermatologic | -5 | -4 |
| Injuries/Adverse Effects: Minor | -- | -1 |
| Injuries/Adverse Effects: Major | 2 | 2 |
| Psychosocial: Time Limited, Minor | 2 | -1 |
| Psychosocial: Recurrent or Persistent, Stable | 1 | -3 |
| Psychosocial: Recurrent or Persistent, Unstable | 13 | 16 |
| Signs/Symptoms: Minor | 3 | 3 |
